# Small Molecules as Toll-like Receptor 4 Modulators Drug and *In-House* Computational Repurposing

**DOI:** 10.3390/biomedicines10092326

**Published:** 2022-09-19

**Authors:** Lucía Pérez-Regidor, Joan Guzmán-Caldentey, Nils Oberhauser, Carmen Punzón, Balázs Balogh, José R. Pedro, Eva Falomir, Alessandra Nurisso, Péter Mátyus, J. Carlos Menéndez, Belén de Andrés, Manuel Fresno, Sonsoles Martín-Santamaría

**Affiliations:** 1 Department of Structural and Chemical Biology, Centro de Investigaciones Biológicas “Margarita Salas”, CSIC, C/Ramiro de Maeztu, 9, 28040 Madrid, Spain; 2 School of Pharmaceutical Sciences, University of Geneva, University of Lausanne, Rue Michel Servet 1, CH-1211 Geneva, Switzerland; 3 Centro de Biología Molecular “Severo Ochoa”, CSIC-Universidad Autónoma de Madrid, 28049 Madrid, Spain; 4 Department of Organic Chemistry, Semmelweis University, H ő gyes E. u. 7, H-1092 Budapest, Hungary; 5 Department of Organic Chemistry, Universidad de Valencia, 46100 Valencia, Spain; 6 Department of Inorganic and Organic Chemistry, Escuela Superior de Tecnología y Ciencias Experimentales, University Jaume I, Av. Sos Baynat, s/n, 12006 Castellón, Spain; 7 Unidad de Química Orgánica y Farmacéutica, Departamento de Química en Ciencias Farmacéuticas, Facultad de Farmacia, Universidad Complutense, 28040 Madrid, Spain; 8 Immunobiology Department, Carlos III Health Institute, 28220 Madrid, Spain

**Keywords:** toll-like receptor 4, innate immunity, drug repurposing, virtual screening, docking

## Abstract

The innate immunity toll-like receptor 4 (TLR4) system is a receptor of paramount importance as a therapeutic target. Virtual screening following a “computer-aided drug repurposing” approach was applied to the discovery of novel TLR4 modulators with a non-lipopolysaccharide-like structure. We screened almost 29,000 approved drugs and drug-like molecules from commercial, public, and *in-house* academia chemical libraries and, after biological assays, identified several compounds with TLR4 antagonist activity. Our computational protocol showed to be a robust approach for the identification of hits with drug-like scaffolds as possible inhibitors of the TLR4 innate immune pathways. Our collaborative work broadens the chemical diversity for inspiration of new classes of TLR4 modulators.

## 1. Introduction

Identification of drug-like molecules with potential therapeutic applications for the treatment of toll-like receptor-related diseases has attracted considerable interest due to their clinical potential. Toll-like receptor (TLR) modulators have the potential to be used with different biomedical applications, especially in the field of infection [1,2], inflammation [3], and autoimmune diseases [2,4], as well as in cancer [5,6,7] and in central nervous system disorders such as Alzheimer’s disease [8]. In particular, TLR4 has recently attracted great attention as a therapeutic target for the discovery of agonist and antagonist drugs for the treatment of a wide range of pathologies [9,10]. TLR4 agonists are useful as adjuvants in cancer immunotherapy and vaccines [11,12]. For example, synthetic nontoxic lipopolysaccharide (LPS) analogs, such as monophosphoryl lipid A derivatives, are components of vaccines for hepatitis B (Fendrix™, GlaxoSmithKline Biologicals SA, Rixensart, Belgium) and cervical cancer (Cervarix™ GlaxoSmithKline Biologicals SA, Wavre, Belgium) [13]. However, even though several inhibitors of the TLR4/MD-2 complex acting on MD-2 can be found in the literature, only a minority of them show sufficiently promising characteristics to become a marketed drug. Eritoran, for example, showed promising results in phase I and II clinical trials, but in phase III, it failed to show better properties than the existing treatments for sepsis [14]. In fact, just a few candidates are currently under clinical development due to the difficulty to find molecules with appropriate physicochemical properties and low toxicity [15]

Therefore, it is imperative to find new chemical entities, not necessarily with an LPS-like structure, such as TLR4 modulators, with good drug-like properties in order to facilitate their development as drugs. There are some small molecules that exemplify this possibility [16]. For example, some pyrimido[5,4-*b*]indoles that have shown to stimulate TLR4 and could potentially be used as adjuvants or immune modulators [17]; synthetic analogs of the natural product euodenine A have exhibited potent and selective agonist activity towards TLR4 [18]; and synthetic peptides able to mimic TLR4/LPS interaction have also been reported [19]. Furthermore, several small non-LPS-like molecules with TLR4 antagonist activity have been developed, such as ethyl 4-oxo-4-(oxazolidin-3-yl)-butenoate derivatives (OSL07) [20], benzothiazole-based inhibitors [21], ethyl phenyl-sulfamoyl cyclohexenecarboxylate derivatives (TAK-242 or resatorvid) [22], and β-amino alcohol derivatives [23]. Interestingly, 6-shogaol has been recently reported as a potential TLR4 inhibitor with a preventive effect on an experimental treatment of knee osteoarthritis [24].

In the context of drug discovery, virtual screening (VS) techniques have already proved to facilitate goal-oriented hit identification, allowing access to a huge number of chemically diverse ligands (from public and commercial databases) with a relatively low cost in terms of time and materials [25,26]. This computational approach has been subject to extensive attention and revision over the years, from the early perspective of being an emerging method [25,26] until the current time where new challenges are faced [27,28,29,30,31,32]. In fact, virtual screening approaches constitute the current strategy in drug design for the identification of novel chemical entities with activity as toll-like receptor modulators [33,34]. However, it could be considered that TLRs are not standard receptors that could be approached following classical strategies in drug design. The complexity of the system and the characteristics of their complexation with pathogen-associated molecular patterns (PAMPs) make them especially difficult to tackle following classical procedures in drug design and discovery. This is why TLRs constitute a special case in this context. Specifically, in the field of TLR4 research, several virtual screening studies have been reported leading to novel TLR4 modulators with drug-like properties, thus overcoming the solubility problems associated with LPS mimetics [34].

On the other hand, despite the enormous effort of time and money spent on research and development, the number of new drugs brought to the market yearly is very low, with strong oscillations [35,36]. Significant investments by pharmaceutical companies for optimizing the drug discovery pipeline have been undertaken, and new techniques such as structure-based drug design, combinatorial chemistry, and high-throughput screening techniques have emerged. Unfortunately, the impact of these innovations has not been as significant as it was expected both in the short and long term [37]. Drug repurposing (also known as drug repositioning, drug redirecting, or drug reprofiling) is a process of discovering new uses outside the scope of the original medical indication for the existing drugs. No traces of this process were found in the literature published before 2004 [38], but it has been gaining an increasing attention within the international drug development community over the last few years and represents a promising new direction [39,40,41,42,43,44]. Different terms are used to describe drug repurposing, but they all mean a way to find new indications for the existing drugs or potential drug candidates, including those in clinical development where the mechanism of action is relevant to multiple diseases: drugs that have failed to demonstrate efficacy for a particular indication during Phase II or Phase III trials but with no major safety concerns; drugs that have been discontinued for commercial reasons; marketed drugs for which patents are close to expiry; and drug candidates from academic institutions and public sector laboratories that have not been fully pursued yet are also taken into account. In this way, drug repositioning represents unique translational opportunities, and it is believed to offer great benefits over de novo drug discovery, reducing the development risks and timeline to potentially 3–12 years [44]. Successful repurposing examples discovered by serendipity are sildenafil, acetylsalicylic acid, and thalidomide [45,46,47].

In this work, we identified novel TLR4 modulators with a non-LPS-like structure by means of virtual screening following a “computer-aided drug repurposing” approach. We screened almost 29,000 approved drugs and drug-like molecules from commercial, public, and *in-house* academia libraries and identified several compounds with TLR4 antagonist activity. Our work opens up opportunities for the development of new chemical classes of TLR4 modulators with therapeutic applications.

## 2. Experimental Section

### 2.1. Computational Methods

#### 2.1.1. Receptors

There are several available 3D structures of TLR4, such as hetero/homodimers and in complex with some ligands (agonists and antagonists) and/or coreceptors [48]. In the case of the agonist conformation of the human TLR4/MD2 (hTLR4/MD-2) monomer complex, 3D coordinates from the hTLR4/MD-2 heterodimer were obtained from the PDB (PDB ID: 3FXI) [49]. In the case of the antagonist conformation, since the full crystallographic structure of the hTLR4/MD-2 complex is not available, a model built by us was used. This model was built using the hMD-2 protein in the antagonist conformation (PDB ID: 2E59) [50] superimposed onto the MD-2 subunit of the agonist full complex (PDB ID: 3FXI chain C) through PyMOL [51]. Furthermore, in order to consider different antagonist conformations of TLR4, we used PDB ID 2E56 (only in the case of the SPECS and Log P 1000 databases).

#### 2.1.2. Databases

Database processing constitutes a fundamental step in VS approaches. It is crucial to generate a proper chemical library with adequate geometries, ionization states, conformations, etc. Good database processing will ensure a rigorous and well-conducted VS, while avoiding high computational costs and identification of unsuitable drug candidates. 

In this work, different commercial, public, and *in-house* databases were used: Log P 1000 [52], SPECS [53], and ZINC [54] as commercial and public databases and a diverse collection of compounds from laboratories of Prof. Péter Mátyus (PM, 1964 heterocyclic compounds selected from the molecule bank of the Department of Organic Chemistry, Semmelweis University (initiated and designed by Péter Mátyus); the listed PM compounds were originally prepared by Andrea Czompa (PM 1090); Paola Bottino (PM 1200); Akos Kocsis (PM 1097, 567); Elias Maccioni (University of Cagliari, PM 810); Judit Kosary (PM 1758, 1779); Klara Czako (PM 1811)), Prof. José Carlos Menéndez (JCM, 68 compounds with quinoline, quinazoline, and acridine structures) [55], Prof. José Ramón Pedro (JRP, 25 compounds, including pyrroles, indoles, and naphthols) [56,57], and Profs. Alberto Marco, Miguel Carda, and Eva Falomir (AM, 85 compounds, including pyrroles, indoles, and naphthols) as *in-house* databases [58]. It is important to mention that, given that paclitaxel had shown antagonistic activity in *h*TLR4, we were prompted to include tubulin binders in our VS approach. We chose a family of compounds analogous to natural products colchicine and pironetin (Figure S5). Regarding their antitumoral activity and their ability to bind to tubulin components and microtubules, paclitaxel is a tubulin-interacting drug that stabilizes microtubules, while colchicine causes disruption of microtubules and pironetin derivatives bind to α-tubulin, inhibiting tubulin assembly. We also included compounds derived from stilbene such as resveratrol since they are studied for their antimitotic properties and their antitumor activity; all of them are included in the AM database. 

#### 2.1.3. Library Preparation

Importation: All the databases were saved as an SD file and imported to Maestro (Schrodinger, version 10.4) [59], which is an all-purpose molecular modeling environment. During the importation process, the chirality and the atom type of each compound were checked. 

Ligand preparation using LigPrep: LigPrep (Schrodinger, version 3.6) [60], a program for preparing all-atom 3D structures of drug-like molecules, was used for many purposes: to refine the geometry of the ligands imported from the databases; to generate accurate, energy-minimized 3D molecular structures; to expand to tautomers, conformations and stereoisomers in order to produce broad chemical and structural diversity from each input structure and predict protonation states. The 3D structures were minimized using OPLS 2005 [61]; to generate ionization states, Epik [62,63,64] was used in order to simulate the physiological pH. In many cases, the compounds contained water molecules or ions; these extra molecules were removed with the Desalt option.

The generated tautomer options were also used in order to generate up to eight tautomers per input structure. Regarding the set stereoisomer options, the choice of retaining the specified chirality to keep this information from the input file and fixing the chirality for the entire calculation was made. The number of stereoisomers generated was limited to 32 per ligand. From a 2D structure, it is not immediately obvious which ring conformations give the lowest energy or are preferred for binding to an active site. Therefore, it was decided to generate one low-energy ring conformation per ligand with LigPrep. The final output was in the Maestro format to keep the total information calculated for all the compounds. For the VS, the compounds were selected according to their molecular weight (300–700 Da) and lipophilicity (4–6) using the property calculation tool from Maestro. 

We considered the following filters:

1. Lipophilicity of the molecules: a maximum logP of 6 was considered taking into account that the natural LPSs and the reported synthetic glycolipids have a very high logP: 29.14 ± 0.83, 14.35 ± 0.73, and 13.53 ± 0.47 for lipid IVa, P01, and ONO-4007, respectively. This limit is within a reasonable margin above the value of 5 according to Lipinski’s rule (oral bioavailability) [65].
2. Molecular weight (MW): we considered a wide range between 300 and 700 Da given the MW of the glycolipids targeting TLR4, with a reasonable margin above the value of 500 according to Lipinski’s rule.
3. pH: only possible tautomers at the physiological pH were considered within a range of 7 ± 0.5.
4. Prediction of favorable binding from at least two docking programs and in two different conformations of TLR4.

#### 2.1.4. Protein Preparation

In the case of the agonist conformation of the TLR4/MD-2 monomer, 3D coordinates from the TLR4/MD-2 heterodimer were obtained from the PDB (PDB ID: 3FXI) [49]. By contrast, in the case of the antagonist conformation, since the full crystallographic structure of the TLR4/MD-2 complex is not available, a model built by us was used [66]. This model was built using the *h*MD-2 protein in the antagonist conformation (PDB ID: 2E59) [25] superimposed onto the MD-2 subunit of the agonist full complex (PDB ID: 3FXI chain C) through PyMOL. Then, coordinates from the TLR4 chain of the 3FXI adjacent to the superimposed MD-2 (PDB ID: 3FXI chain A) and the superimposed MD-2 in the antagonist conformation were retained, forming the TLR4/MD-2 monomer in the antagonist conformation. Finally, both the agonist and antagonist structures were subjected to 10,000 cycles of the steepest descent energy minimization under an AMBER force field via Maestro. Furthermore, PDB ID 2E56 was used to consider different antagonist conformations of MD-2.

#### 2.1.5. Receptor Grid Preparation

GLIDE: For preparing the receptor grids for the two protein conformations, GLIDE (Schrodinger, version 6.9) was used [67,68,69]. All the parameters of the software were kept at their default values. We only determined where the scoring grids would be positioned and their sizes. The coordinates of the box were set up to fully contain *E. coli* LPSs. GLIDE uses two “boxes” that can be parametrized to organize the calculation: the inner box, which can be monitored in the advanced panel and where the ligand center is allowed to move during the site point search, and the outer box, which is the box within which all the ligand atoms must be contained. Its size is function of the inner box, and the inner box has to be included within the outer box. For the inner box, the center was set up at residue serine 120 and the lengths of the boxes for both protein conformations were as follows: 33 Å for X, 40 Å for Y, and 35 Å for Z. For the outer box, 10 Å were added to the dimensions of the inner box (43 Å for X, 50 Å for Y, and 45 Å for Z).

FLAP [70,71], AutoDock [72], and VINA [73]: As the receptor grids were already set up with GLIDE, the same grids were chosen for these programs. The GLIDE coordinates were kept for VINA but were converted into AutoDock coordinates using a scaling calculation tool. In the case of FLAP, the pockets of MD-2 were identified and defined by the FLAP’s pocket search algorithm.

#### 2.1.6. Docking

##### Structure-Based Virtual Screening (SBVS) with FLAP

The FLAP software explicitly distinguishes between the so-called SBVS method and docking [70,71]. While in FLAP docking is primarily used for pose prediction and a more precise quantification of binding energies, SBVS is a tool for large-scale virtual screening. Even though docking is often used as a structure-based virtual screening technique, [74] the term SBVS hereafter refers only to the FLAP’s corresponding screening program. 

The SBVS program first creates MIFs of the receptor’s binding site. During screening, the MIFs of the ligand are compared with those of the binding site. Time-consuming calculations describing each atom–atom interaction are not needed here. One downside of this method is that there is no energy penalty for atom clashing with the target. In some scenarios, however, this might even be an advantage since it overcomes the rigidity of the target to some extent. 

The SBVS in FLAP was performed on a 3D structure of the human coreceptor MD-2. The structure was obtained from the PDB (PDB ID: 2E56) [50], and the MOE software was used to prepare the protein by removing water molecules, adding hydrogens and the missing atoms and side chains [75]. The optimized structure was loaded into FLAP and the Search for Pockets function was used to define the binding area. The results were then treated similarly to the LBVS approach.

##### SBVS with GLIDE

The molecules were subjected to grid-based ligand docking with energetics (GLIDE, Schrodinger, version 6.9) [67,68,69] using the Virtual Screening Workflow protocol. It is designed to run an entire sequence of jobs for screening large collections of compounds against one or more targets. However, as the compounds and the grids had already been prepared, in this case, only the docking steps of the program were used. The compound files and the receptor grid files were imported into the Virtual Screening Workflow program. Regarding the docking step parameters, Epik state penalties for docking were used, and the nonpolar part of the ligand potential was softened by scaling the van der Waals radii of ligand atoms with small partial charges. To do so, the scaling factor was 0.80, and the partial charge cutoff was 0.15. The full workflow includes three docking stages, each step differing from the preceding step in the amount of time taken to dock each molecule and the scoring system used to evaluate each pose. The first stage performs HTVS (high-throughput virtual screening) docking. The ligands that are retained are then passed to the next stage, which performs SP (standard precision) docking. 

The survivors of this stage are passed onto the third stage which performs XP (extra precision) docking, a more powerful and discriminating procedure.

The dock flexibility method was used for the HTVS, SP, and XP docking allowing us to penalize nonplanar conformations for amide bonds. Post-docking minimization was also performed, as well as constraints for the docking stages. One pose per compound state was generated and 100% of the best compounds that passed the HTVS, SP, and XP docking were kept. For the HTVS and SP docking, all the states were retained, but only the best-scoring state was retained for the XP docking.

##### SBVS with AutoDock4 and AutoDock VINA

Docking was also performed independently with both AutoDock [72] and VINA [73]. In AutoDock, the Lamarckian evolutionary algorithm was chosen and all the parameters were kept default except for the number of genetic algorithm (GA) runs which was set to 50 to sample more docked poses. VINA (Vina Is Not AutoDock) uses an iterated local search global optimizer [73] based on the Broyden–Fletcher–Goldfarb–Shanno (BFGS) algorithm which approximates Newton’s method, and the number of docking poses was set to 20, which is the maximum for the program. The TLR4/MD-2 receptors were kept rigid and the ligands were set to be partially flexible (i.e., maximum of 32 dihedral angles) for AutoDock and totally flexible for VINA.

##### Ligand Redocking Using GLIDE

The shortlisted molecules were submitted to a redocking procedure using GLIDE. All the parameters were kept as mentioned in the docking paragraph using GLIDE, except for the docking poses which were set to 50 per molecule.

##### Molecular Redocking Using FLAP

FLAP implements a fragmentation-based docking algorithm called FLAPdock, which works as follows. MIFs are calculated for the target-binding site in a similar manner to the SBVS approach but with more points to describe the site in more detail (reference manual for FLAP 2.0, © 2014 Molecular Discovery Ltd., Hertfordshire, UK). A set of ligand conformations is generated using a stochastic search and a customized implementation of the MM3 force field [76] with a cutoff of 30 kcal/mol^−1^ to remove high energy and duplicate conformations. The ligands are then split into fragments with only 1–3 rotatable bonds. For each fragment conformation, GRID MIFs are calculated. The first fragment is docked into the binding site and the best-scoring solutions, according to the global S-Score, are retained for the next iteration. In the next step, the next fragment is attached to the first one and scored in the same way. The S-Score is a scoring function that includes terms from the GRID MIF similarities (hydrogen bonding and hydrophobic interactions as well as shape matching), Lennard–Jones and electrostatic interactions. 

It was validated, amongst other targets, on those of the Astex and DUD datasets [68,77,78]. In each iteration, the best-scoring solutions are kept and filtered by RMS clustering. Once the reconstruction of the ligand has finished, the final pose can be optionally optimized by minimization, and the final score is recalculated. The benefit of FLAPdock in comparison with the SBVS method lies in the more detailed chemical interactions that are considered for docking and the consideration of steric clashes that are not regarded in the SBVS method. In order to obtain score reference values, a set of known MD-2 inhibitors was docked, followed by the docking of compounds from the Log P 1000 and the SPECS dataset.

### 2.2. Biological Characterization

HEK-Blue TLR4 assay: HEK-Blue TLR4 cells (InvivoGen, Toulouse, France) and parental cell line HEK-Blue Null 2 (InvivoGen) were used to test the agonist or antagonist effect of different compounds. This cell line expresses TLR4, MD-2, and CD14 and does not express any other TLRs. The activation of TLR4 leads to the expression of SEAP, a protease that enzymatically hydrolyzes the molecule present in the medium. The amount of hydrolysis can be measured using colorimetric methods. These cells were cultured according to the manufacturer’s instructions. Briefly, the cells were cultured in a high-glucose DMEM supplemented with 10% fetal bovine serum (FBS), 1% glutamine, 1% penicillin/streptomycin, 1× Normocin (InvivoGen). The experiments were performed when 70–80% of confluence was reached. The cells were detached by the use of PBS and tapping the flask and the cell concentration was estimated using Trypan Blue (Sigma-Aldrich, St Louis, MO, USA). Four different compound concentrations were used: 0.1, 1, 5, and 10 μg/mL. Twenty microliters of the diluted compound were added to a 96-well plate in triplicate (three wells for each concentration), seeded in a multiwall plate at a density of 2 × 10^4^ cells/well in 200 µL. LPSs were used as the positive control (final concentration of 20 ng/mL) and 1× PBS was used as the negative control. The cells were detached using 4 mL of PBS, and a 140,000 cells/mL solution was prepared using the HEK-Blue Detection medium. One hundred eighty microliters of this solution were added into each well (25,000 cells/well). After a 30 min incubation, 20 µL of the LPS solution were added to each well (final LPS concentration: 20 ng/mL) (LPSs were diluted in PBS as well). The plates were incubated for 16 h in the dark at 37 °C, 95% humidity, and 5% CO_2_ and then the plate reading was assessed using a spectrophotometer at 620 nm. The results were normalized with the positive control (LPSs alone) and expressed as the mean percentage ± SD of at least three independent experiments.

TNF-α detection: An adherent murine macrophage cell line J774.2 was grown in 75 cm^3^ cell culture flasks in Dulbecco’s modified Eagle’s medium supplemented with 5% fetal bovine serum and 1% penicillin/streptomycin. Approximately 2 × 10^6^ cells were plated in individual wells of a 12-well plate. The cells were stimulated with LPSs from *Escherichia coli* (Sigma-Aldrich) at a final concentration of 2 ng/mL. The respective compounds were added to 1 ng/mL of LPSs at 5 µg/mL. As the positive control, LPSs (1 ng/mL) with DMSO were used. Two wells per compound and control were used. The plates were incubated for 24 h at 37 °C and 5% CO_2_. The cell supernatants collected after 24 h stimulation assays were analyzed for TNF-α. Commercial enzyme-linked immunosorbent assay kits were used (Mouse TNF-α DuoSet ELISA, R&D Systems). ELISA was performed in 96-well plates, and the plates were read at 450 nm in a microplate reader.

## 3. Results and Discussion

### 3.1. Searching for TLR4/MD-2 Modulators: Virtual Screening

#### General Overview of the Virtual Screening Protocol

**Figure 1:**
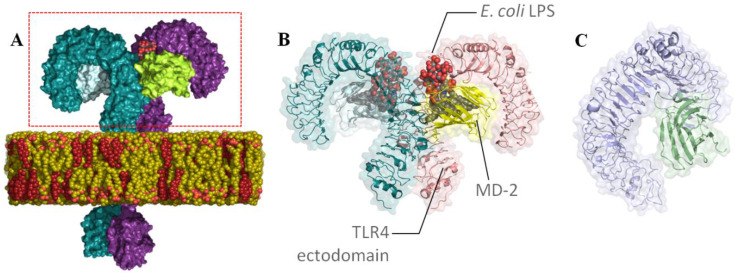
(**A**) Full-atom 3D model of the agonist LPS-bound TLR4 dimer in a membrane environment [81]; ectodomain is framed in a red box. (**B**) X-ray structure of the TLR4/MD-2 dimer in complex with *E. coli* LPSs (from PDB ID 3FXI). (**C**) Three-dimensional model of TLR4/MD-2 in the antagonist conformation [66].

TLR4 is specialized in the recognition of lipopolysaccharides from Gram-negative bacteria through the extracellular domain (ectodomain) with the participation of an essential coreceptor, myeloid differentiation factor 2 (MD-2) (Figure 1) [66,79,80,81]. The LPS fatty acid chains are inserted into the MD-2 pocket while the oligosaccharide binds to TLR4 and the partner TLR4. Dimerization of the ectodomain promotes the TLR4/MD-2 dimerization at the intracellular site and recruitment of the binding of adaptor proteins that finally triggers the activation of downstream signaling and the inflammatory response (Figure 1) [79,80,81].

**Figure 2:**
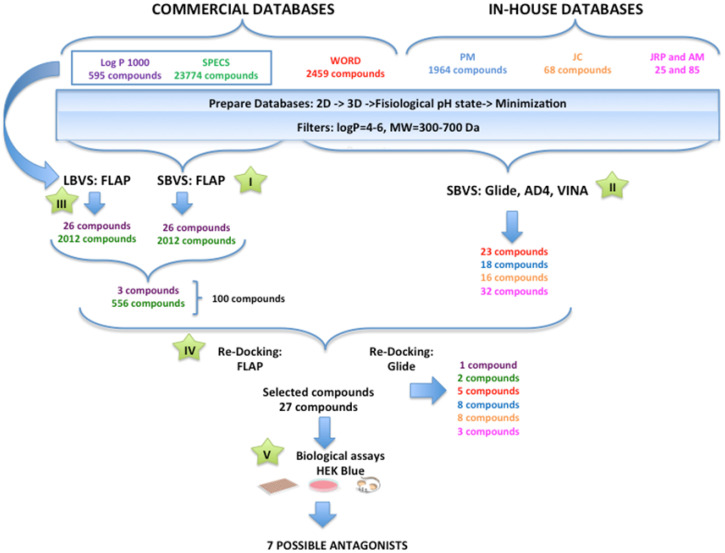
Flowchart of the virtual screening, redocking, and biological assay protocol.

Molecular docking screening was performed against the different databases based on both the agonist conformation of the *h*TLR4/MD-2 complex from PDB ID 3FXI and our modeled antagonist conformation of the *h*TLR4/MD-2 complex [66]. Ligand-based (LBVS) and structure-based (SBVS) virtual screening were carried out following the protocols shown in Figure 2. The Log P 1000 and SPECS databases were submitted for LBVS (step III) and SBVS (step I) with the FLAP tool. WORLD (subset of the ZINC database) and *in-house* databases were submitted for SBVS with the combined GLIDE/AutoDock/VINA approach (step II). The resulting screened compounds (189 in total) were redocked (step IV) by means of FLAP and GLIDE, finally yielding 27 compounds that were experimentally tested (step V). Seven compounds were identified as TLR4 antagonists.

### 3.2. Performance of the Virtual Screening Study

#### 3.2.1. Step I. Structure-Based Virtual Screening (SBVS) with FLAP

The FLAP’s SBVS method was used to perform target-based virtual screening on the TLR4/MD-2 receptor with the SPECS and Log P 1000 databases. As the benchmark, the method was applied initially to a set of reference compounds: small molecules with known activity as TLR4 antagonists. The results are shown in Table 1 with the ligands ranked according to their Glob-Sum score. This score is a global similarity score calculated by summing the four single contributions: shape (H), hydrogen bond acceptor (N1), hydrophobic (DRY), and hydrogen donor acceptor (O) descriptors.

The Glob-Sum scores obtained for the known ligands were used as reference values to identify potential hits. The highest score was obtained by paclitaxel (Glob-Sum = 3.245) which was then used as a cutoff value for the screened unknown ligands. From the Log P 1000 and SPECS libraries, 26 and 2012 compounds were obtained, respectively, having a score equal to or higher than 3.245 (Figures S1 and S2). The highest contribution to the global score was given by the hydrophobic score which can easily be explained by the high hydrophobicity of the target pocket and the screening model that is obtained from it.

#### 3.2.2. Step II. Structure-Based Virtual Screening (SBVS) with GLIDE, AutoDock, and VINA

The WORLD database from ZINC and *in-house* databases (PM, JCM, JRP, and AM) were docked into both the agonist and antagonist protein conformations using three docking programs, GLIDE, AutoDock, and VINA, to avoid the limitation of one scoring function. The receptor grid was set up in order to fully contain the *E. coli* LPSs, allowing small molecules to interact with the entire MD-2 pocket, as well as its rim and its entrance (see the Materials and Methods). During the docking process, all the ligands were kept to facilitate visual inspections, comparisons, and selections between the three docking programs. Fifty poses per ligand were generated with AutoDock, 20 poses per ligand—with VINA (which is the maximum for the program), and only one pose per ligand was generated with GLIDE using the HTVS, SP, and XP protocols in order to also facilitate the comparisons, choosing GLIDE as the main docking software. For the docking program validation analysis either with GLIDE, AutoDock, or VINA, the scoring results for all the compounds were consistent and correlated to each other. However, the correlation between AutoDock and VINA was stronger than between GLIDE and AutoDock or VINA. The docked compounds, as well as all their corresponding predicted binding poses, were visually analyzed to detect any computational errors. The docking scores, defined by the average score of all the poses from one ligand for each docking program, were analyzed. The selected screened compounds from each docking program were visually inspected, and those binding outside MD-2 were discarded. Finally, only the top 10% in the case of the WORLD and PM databases and 20% from the JCM, JRP, and AM databases were kept. Among them, 89 compounds were ranked at the top by at least two docking programs and were predicted to bind into one (at least) of the two TLR4 conformations (agonist/antagonist). These 89 compounds were then submitted to the following analysis step (step IV), redocking with AutoDock and GLIDE.

#### 3.2.3. Step III. Ligand-Based Virtual Screening (LBVS) with FLAP

Only in the case of the Log P 1000 and SPECS commercial databases we also performed LBVS with the FLAP tool [39]. The FLAP’s LBVS method uses a common reference framework to align a set of candidate molecules to the template binder to find the optimal overlap according to the GRID molecular interaction fields (MIFs) [39]. The similarity between the fields is quantified using the Tanimoto coefficient. In the output table, the user can see the individual scores obtained by the single MIF contributions (Glob-Prod), as well as the global score representing the sum (Glob-Sum) for each compound. For the LBVS with FLAP, a set of known active antagonists of MD-2 was built based on a literature search (Figure S3). The two datasets Log P 1000 and SPECS were screened on each known active compound separately and ranked by their obtained Glob-Sum scores. LBVS was performed individually by using the 14 known MD-2 ligands as templates for the screening. The best ranked results are shown in Table 2. The 2D representations of the compounds of Log P 1000 and SPECS can be found in Figures S1 and S2, respectively.

The results shown in Table 2 indicate that for each of the known active compound, the best-scoring SPECS compound scored higher than the best-scoring one from the Log P 1000 database. This could be explained by the sole fact that the SPECS set contains a much higher number of compounds than Log P 1000. Consequently, the probability of finding a well-scoring compound is higher.

Regarding the single contributions of the four similarities, the shape similarity (H) seems to have the highest impact on the global score in most of the cases. In four cases (6-shogaol, CAPE, curcumin, C34), the hydrogen bond acceptor and in one case the hydrogen bond donor (cinnamaldehyde) similarities made the biggest contribution to the global score. All the five compounds are from the SPECS set. The reason why the influence of hydrophobic (DRY) similarity is comparatively low might be the relatively small size of the compounds. While strong hydrogen bond similarities can be derived from single donor or acceptor atoms, the hydrophobic potential needs larger nonpolar surfaces to show a strong impact. 

#### 3.2.4. Step IV. Redocking with FLAP and GLIDE

To better understand the interactions of the potential inhibitors retrieved by LBVS and SBVS with TLR4/MD-2, a molecular redocking approach was carried out. In order to narrow down the number of compounds to dock, only molecules were selected which obtained a good score in the LB and SBVS approaches. From the SBVS, in total, 2038 compounds (26 from Log P 1000 and 2012 from SPECS) obtained a score higher than the cutoff value of 3.245. Since an analogous cutoff value was not available for the LBVS approach, the same number of compounds was chosen here, i.e., the top ranked 26 and 2012 compounds from Log P 1000 and SPECS, respectively. 

From the Log P 1000 set, three common compounds were found in the top ranks of both the LBVS and SBVS, while SPECS shared 556 top-ranked compounds. This total number of 559 compounds still seemed large considering the time-consuming FLAP docking program. For this reason, only the top 100 highest-scoring ligands were taken for the final redocking (step IV). This selection procedure was found to be in agreement with examples from the literature [82].

The screened compounds from the above steps (189 compounds in total) were re-docked by means of FLAP and GLIDE, finally leading to the selection of 27 compounds. This selection was based on (i) the agreement in the most probable clusters from both programs and (ii) the visual analysis of the best clusters from both docking programs with special attention to the ligand/receptor interactions (discussed below). 

The finally selected compounds were as follows: one compound from Log P 1000 (ID-5382), two compounds from SPECS (AG-690/11203225 and AF-399/1512855) (Table S1), five compounds from the WORLD database (compounds 146, 157, 177, 179, and 208) (Table S2), eight compounds from PM (PM1097, PM1811, PM1779, PM567, PM1090, PM810, PM1758, and PM1200) (Table S3), eight compounds from JCM (MS14, MS20, MS21, MS32, MS35, MS40, MS45, and MS49) (Table S4), and three compounds from JRP–AM (JRP07, JRP07p, and JRP10) (Table S5).

The three ligands from the Log P 1000 and SPECS datasets were initially screened with FLAP and had an S-score equal to or higher than the threshold of 1.074 obtained by the best-scoring known inhibitor sulforaphane. The compounds, their 2D description, and the respective scores are listed in Table S1. The highest-scoring compound was ID-5382 from the Log P 1000 set, with an S-score of 1.231. The two compounds of the SPECS set AG-690/11203225 and AF-399/15128553 obtained the scores of 1.114 and 1.074, respectively. 

**Figure 3:**
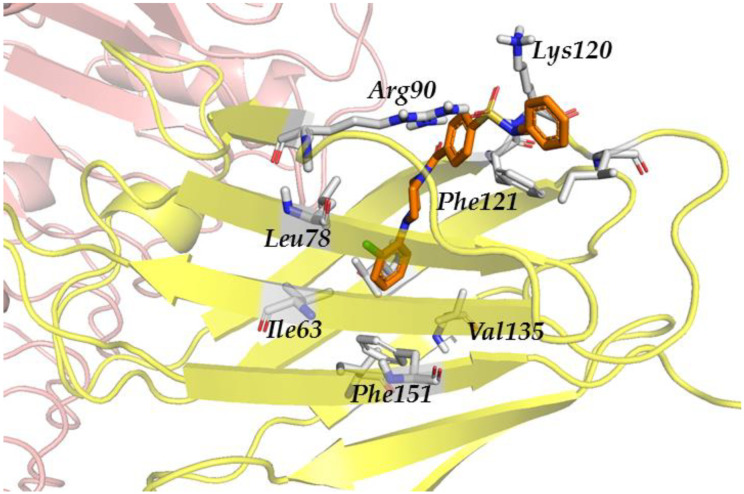
Redocked ID 5382 (orange sticks) in TLR4/MD-2. MD-2-interacting residues are highlighted with grey sticks. TLR4 is depicted in pale pink, MD-2—in yellow.

When studying the docked poses, in all the three cases (ID-5382, AG-690/11203225, and AF-399/15128553), the docked ligand was located at the entrance of the hydrophobic pocket of MD-2, adopting similar poses. The principal interactions were hydrophobic and polar. The three compounds showed polar interactions with Arg90 and Lys122. The hydrophobic interactions were more widespread and not with the same set of amino acids for the three compounds. It could be observed that compound ID-5382 was in close contact with hydrophobic residues Ile46, Leu63, Leu78, Phe121, Ile124, Val135, and Phe151 (Figure 3). Compound AG-690/11203225 interacts with residues Ile52, Phe76, Leu78, Ile80, Val82, Glu92, Phe121, Ile124, Val135, and Ile153. Finally, the hydrophobic interactions of AF-399/15128553 were established with the Ile46, Leu61, Ile80, Val82, Leu87, Phe121, Ile124, Tyr131 and Phe151 side chains.

Residues Arg90 and Lys122 from MD-2 are able to build salt bridges with compounds ID-5382 (Figure 3) and AG-690/11203225 due to their sulfonyl groups. This would explain why the polar score is significantly higher for these two ligands than for compound AF-399/15128553 which possesses no sulfonyl groups. The latter one only forms hydrogen bonds between the Arg90 and Lys122 side chains and the basic nitrogen atoms from the triazole ring. Interactions with Cys133, such as those reported in the literature [83], could not be observed due to the inability to predict/model covalent bonds with FLAP. 

From the JRP–AM database, the selected compounds established stacking interactions with Phe76, and CH–π interactions were observed with the side chains of Cys133, Phe151, Phe104, and Leu61. The other interactions observed were hydrophobic, with residues Val24, Ile32, Ile44, Val48, Ile52, Leu78, Ile80, Ile94, Ile117, Phe119, Val135, and Ile153.

**Figure 4:**
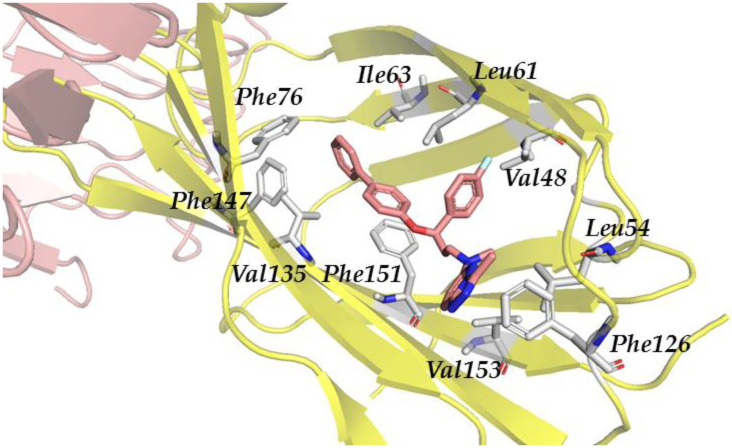
Redocked PM1090 (salmon sticks) compound in TLR4/MD-2. TLR4 is depicted in pale pink; MD-2—in yellow. MD-2 interacting residues are highlighted with grey sticks.

Regarding the compounds from the PM database, they established π–π interactions with Phe104 and Phe151, as well as CH–π interactions with Phe76 and Phe121 (Figure 4). The other interactions observed were hydrophobic, with Ile32, Ile52, Leu61, Ile117, Val135, leu149, and Ile153.

**Figure 5:**
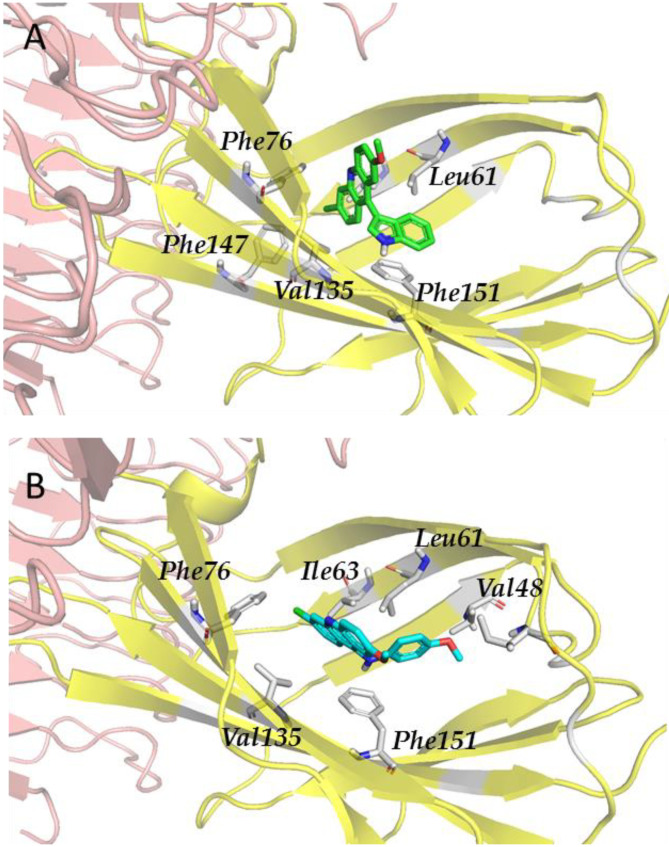
Redocked MS-21 ((**A**), green sticks) and MS-32 ((**B**), cyan sticks) in TLR4/MD2. TLR4 is depicted in pale pink; MD-2—in yellow. MD-2 interacting residues are highlighted with grey sticks.

Finally, in the case of the compounds from the JCM database, the principal interactions observed were as follows: CH–π interactions between both sides of the tricycle and the side chains from the Ile32, Ile52, Leu61, Leu63, Ile94, and Val135 residues, edge-to-face interactions between the tricycle and side chains from Phe76 and Phe147, and π–π or edge-to-face interactions between the aromatic ring attached to the tricycle and the Phe151 side chain (Figure 5).

In summary, it is possible to say that for the Log P 1000, SPECS, WORLD databases and the *in-house* databases (JCM, PM, and JRP–AM), the docked ligands are predicted to bind at the entrance of the hydrophobic cavity of TLR4/MD-2 in similar docked poses. The principal interactions are hydrophobic ones within the inner region of MD-2 and polar ones at the rim of MD-2. Most compounds also show polar interactions with Arg90 and Lys122. Hydrophobic interactions, however, are more widespread and not with the same set of amino acids for all the compounds. To note, some of these interactions have been suggested to be key interactions for the reported TLR4/MD-2 ligands. For example, Arg90 is assumed to participate in interactions with sulforaphane, JTT705, isoxanthohumol, isoliquiritigenin, CAPE, and JSH, while Lys122 interacts with OSL07 and cinnamaldehyde; the Ile80 side chain interacts with xanthohumol, JSH, OSL07, cinnamaldehyde, and 6-shogaol; side chains from Phe121 and Ile124 are also reported as involved in the key interactions with many reported ligands either through π–π or CH–π interactions [33,34].

From all the screening structures that came after the virtual screening protocol, we can extract a common scaffold: two hydrophobic moieties separated by a polar linker. The large hydrophobic part occupies the hydrophobic MD-2 cavity, while the small one is placed in the same hydrophobic region where one of the lipid A alkyl chains is also located in the TLR4/MD-2 X-ray crystallographic structure (PDB ID: 3FXI). The key interactions are those established with residues Arg90 (capable of forming ionic interactions and hydrogen bonds), Phe121 and close Phe126 (capable of forming π–π or CH–π interactions), and Tyr131 (also capable of establishing hydrogen bonds). These interactions were common for all the compounds and conferred them the highest predicted binding energy among all the screened compounds. The polar linker interacts with the positively charged amino acids, Arg90 and Lys122, at the entrance region of the pocket which have already been shown to be important for the binding of the reported active compounds [34,84]. Furthermore, all these screened compounds share a relatively high lipophilicity.

Virtual screening of the WORLD subset from the ZINC database identified five compounds outperforming the remaining ones (Table S2, compounds 146, 157, 177, 179, and 212). Surprisingly, compared to the previous analysis conducted only with the best GLIDE pose, compounds 157 and 212 did not show good results in the last analysis. Indeed, having a wider number of poses in GLIDE permitted to observe for these two compounds that the first pose was not part of the most probable cluster, or any cluster at all, for both conformations. Moreover, it was shown that the most probable clusters for these two compounds were ranked in a low-energy position and with a medium total percentage of interaction against the main residues. Compound 208, previously identified in the first analysis as having a good cluster position, was observed to have medium total percentage of interaction against the main residues. Compounds 157, 208, and 212 were kept as a query for a future structure similarity search.

In ascending order of better predicted binding, compounds 146, 177, and 179 outperformed all the other compounds. Compounds 146 and 177, already revealed by the first cluster analysis, showed having in each pose interactions with almost all the main residues. Moreover, in around 50% of the poses, they were able to make two hydrogen bonds simultaneously, and in around 70% of the poses—two salt bridge interactions simultaneously. Compound 179 was predicted to have the highest affinity potential with all the main residues. It interacted with all the main residues with high affinity, making in 80% of the poses up to three hydrogen bonds and a salt bridge in 50% of the poses. Compounds 146, 177, and 179 were also kept as queries for a future structure similarity search. 

Compound 146 is known as diphenoxylate. It is a meperidine congener used as an antidiarrheal, usually in combination with atropine. At high doses, it acts like morphine. Its unesterified metabolite difenoxin has similar properties and is used similarly. It has little or no analgesic activity because it does not cross the blood–brain barrier. According to DrugBank, it is categorized as an analgesic, opioid, anti-peristaltic agent, alimentary tract and metabolism agent, antidiarrheal, intestinal anti-inflammatory/anti-infective agent, and anti-propulsive. Because TLR pathways can be related to inflammatory and microbial pathologies, it is conceivable that diphenoxylate could have a certain affinity for TLR4. It has also been shown that diphenoxylate can regulate NF-κB [85], a protein present downstream in the TLR pathway. Moreover, some studies have proven the binding between morphine and TLR4 [86,87,88], suggesting a conceivable effect of diphenoxylate in TLR4 as well.

Compound 177 is known as Ono-Rs 411, or pranlukast. It is a cysteinyl leukotriene receptor 1 antagonist. It antagonizes or reduces the bronchospasm caused, principally in asthmatics, by an allergic reaction to accidentally or inadvertently encountered allergens. It is classified as an anti-asthmatic agent, respiratory system agent, drug for obstructive airway diseases, leukotriene receptor antagonist, cytochrome P-450 CYP2C9 inhibitor, cytochrome P-450 CYP2C9 inducer, and CYP3A4 inhibitor. Besides, some studies have shown that pranlukast can inhibit NF-κB activation [89,90]. It has also been shown that it indirectly induces cytoplasmic membrane depolarization of Gram-negative bacteria, promoting *E. coli* outer membrane detachment [91], releasing microbial products that are recognized by TLR4.

Compound 179 is known as vemurafenib, a V600 mutant BRAF enzyme inhibitor for the treatment of late-stage melanoma [92]. Vemurafenib inhibits the active form of the kinase [93,94], firmly anchoring itself in the ATP-binding site. By inhibiting only the active form of the kinase, it selectively inhibits the proliferation of cells with unregulated BRAF, normally those that cause cancer. It is classified as an antineoplastic agent, protein kinase inhibitor, antineoplastic and immunomodulating agent, cytochrome P-450 CYP1A2 inhibitor, cytochrome P-450 CYP1A2 inducer, CYP2D6 inducer, CYP2D6 inducer (strong), and CYP3A4 inhibitor. To date, it has been shown that TLR4 and its signaling pathway promote migration of human melanoma cells [95,96], but no studies showing a direct effect of vemurafenib on TLR4 have been conducted yet.

#### 3.2.5. Step V. Biological Testing

After the identification of possible TLR4 binders, we tested them in HEK-Blue^TM^ cells transfected with *h*TLR4 to check their ability to act as TLR4 agonists or antagonists and in J744 macrophage cells to check their ability to decrease TNF-α secretion.

**Figure 6:**
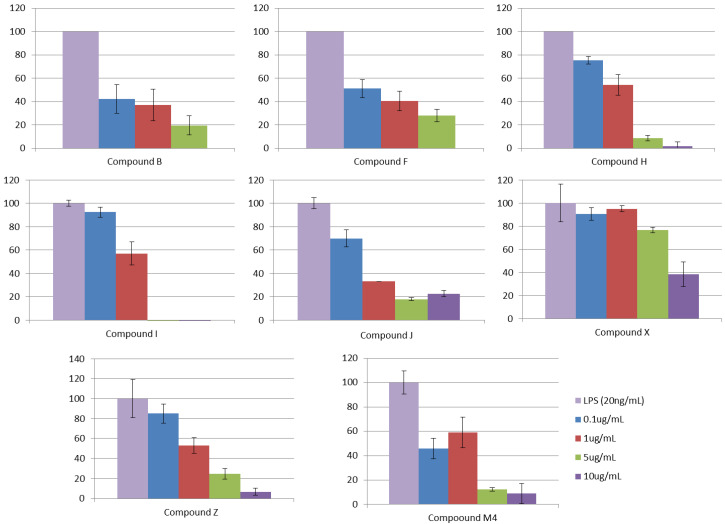
HEK-Blue^TM^ treatment with increasing concentrations of compounds, stimulated with LPSs from *E. coli*. The results are expressed in the percentage of TLR4 activation (positive control: *E. coli* LPSs, 20 ng/mL).

The ability of the ligands to interfere with LPS-triggered TLR4 activation in HEK-Blue *h*TLR4 cells model was investigated. This HEK293 cell line is stably transfected with human TLR4, MD-2, and CD14 genes. In addition, HEK-Blue^TM^ cells express a secreted alkaline phosphatase (SEAP) produced upon activation of NF-κB. LPS binding activates TLR4 and NF-κB, leading to SEAP secretion, which is detected by an alkaline phosphatase substrate in cell culture media (Figure S4). In this assay, the HEK-Blue^TM^ cells were treated with increasing concentrations of synthetic molecules and then stimulated with LPSs from *E. coli* (20 ng/mL). The results were normalized to activation by LPSs alone and expressed as the mean percentage ± SD of three independent experiments. The screened 27 compounds were tested and, from them, compounds B (ID-5382), F (MS21), H (MS32), I (MS35), J (MS45), X (PM1090), Z (PM1200), and M4 (179) inhibited TLR4 activation in a dose-dependent way (Figure 6 and Table 3). As the negative control, compounds were tested in a null cell line (InvivoGen) transfected with the same plasmids as the HEK-Blue^TM^ cells but without the TLR4, MD-2, and CD14 genes, and no effect was observed.

**Figure 7:**
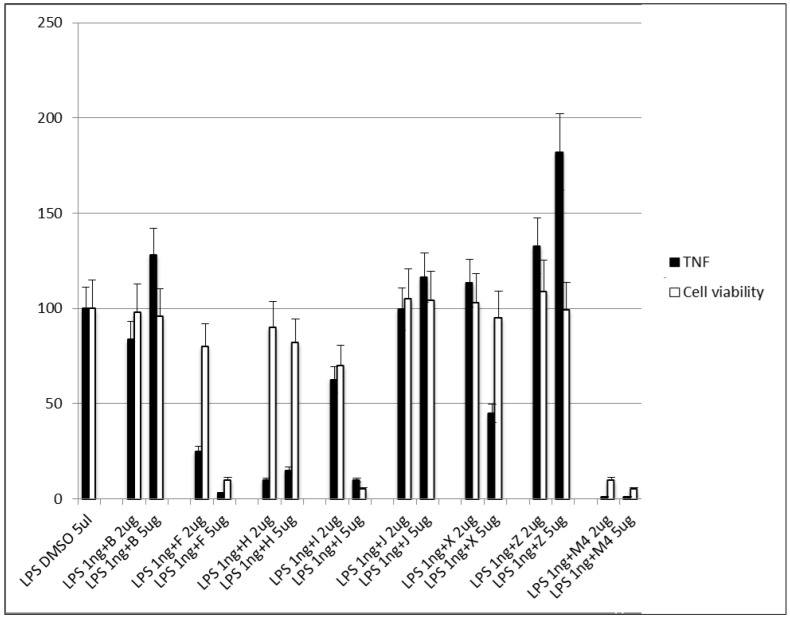
Expression of TNF-α secretion (black bars) and cell viability (white bars) in the J744 cell line upon treatment with the screened compounds B, F, I, J, X, Z, and M4. The results were normalized with the positive control (LPSs alone) and expressed as the mean percentage ± SD of at least three independent experiments.

We also tested the activity of these compounds on J744 macrophage cells in order to detect the amount of TNF-α secreted by the cells in the presence of the compounds. Moreover, in order to test the toxicity of the selected compounds, MTT assay was performed on the same cell line (Figure 7). Compound H showed a strong TNF-α production inhibition at both concentrations, and no significant cytotoxicity effect was observed (cell viability was maintained, Figure 7). These results make compound H one of the most promising scaffolds. Compound F also showed an inhibitory activity at 1 µg/mL, but high levels of cytotoxicity were observed at 5 µg/mL (cell viability decreased, Figure 7). On the other hand, compound X markedly inhibited the production of TNF-α at 5 µg/mL, while no cytotoxic effect was observed at both concentrations (viability was maintained, Figure 7). Compounds B, J, I, and Z did not show any inhibitory effect, and compounds I and M4 showed high cytotoxicity even at 1 µg/mL.

## 4. Conclusions

In this work, we applied virtual screening and computational repositioning strategies for the discovery of novel non-LPS-like TLR4 modulators. Our computational protocol made use of different conformations of TLR4/MD-2 and included ligand-based and structure-based virtual screening and deep ligand/receptor analysis. Our protocol showed to be a robust approach for the identification of eight non-LPS-like compounds with the TLR4/MD-2 antagonist activity. Compounds B (ID-5382), F (MS21), H (MS32), I (MS35), J (MS45), X (PM1090), Z (PM1200), and M4 (sorafenib) inhibited TLR4 activation in a dose-dependent manner as putative of TLR4 modulators. In addition, compounds F and H showed antagonist activity in the J744 cell line with no signs of cytotoxicity. The computationally identified hits represent interesting non-LPS-like scaffolds for a new class of possible inhibitors for the TLR4/MD-2 complex. We also showed the molecules identified by computational screening from the different chemical libraries studied, commercial and public databases, and from the academia. We aimed to share with the scientific community the chemical identity of the compounds resulting from our collaborative work in order to serve as inspiration for future design.

## Figures and Tables

**Table 1:** Reference antagonists of MD-2 ranked by the Glob-Sum score obtained from structure-based virtual screening (SBVS) in descending order.

Antagonist	Glob-Sum	Antagonist	Glob-Sum
Paclitaxel	3.245	6-Shogaol	2.498
JSH	2.714	Isoxanthohumol	2.465
Curcumin	2.695	Isoquiritigenine	2.239
**1D10G**	2.669	Cinnamaldehyde	2.179
**CAPE**	2.621	C34	2.136
**Xanthohumol**	2.611	OSL7	1.799
**JTT705**	2.513	Sulforaphane	1.707

**Table 2:** Best-ranked compounds from LBVS of the Log P 1000 (blue) and SPECS (white) sets for each template (reported MD-2 ligands). The Glob-Sum score is a global similarity score calculated by summing the four single contributions: shape (H), hydrogen bond acceptor (N1), hydrophobic (DRY), and hydrogen donor acceptor (O) descriptors.

Template	Compound	Glob-Sum	H	N1	DRY	O
**6-Shogaol**	152	1.326	**0.663**	0.508	0.224	0.239
	481	1.742	0.598	0.271	0.254	**0.702**
**Xanthohumol**	568	1.269	**0.699**	0.283	0.340	0.124
	19,907	1.912	0.703	0.368	0.508	0.359
**Paclitaxel**	383	0.847	**0.505**	0.175	0.134	0.337
	20,513	1.022	**0.565**	0.171	0.105	0.321
**1D10G**	368	1.152	**0.579**	0.203	0.181	0.310
	20,700	1.857	**0.654**	0.306	0.260	0.734
**JSH**	492	1.165	**0.598**	0.359	0.229	0.144
	21,315	1.421	**0.515**	0.371	0.304	0.329
**Isoliquiritigenin**	42	1.181	**0.637**	0.364	0.195	0.010
	120	1.706	**0.750**	0.431	0.343	0.294
**Isoxanthohumol**	138	1.054	**0.638**	0.234	0.308	0.010
	28	1.493	**0.634**	0.430	0.304	0.305
**CAPE**	575	1.149	**0.625**	0.242	0.181	0.243
	22,298	1.528	0.587	0.230	0.159	**0.700**
**Curcumin**	548	1.041	**0.631**	0.242	0.204	0.010
	23,010	1.562	0.519	0.264	0.173	**0.623**
**Sulforaphane**	46	1.104	**0.650**	0.361	0.166	0.000
	3203	1.184	**0.684**	0.296	0.000	0.000
**Cinnamaldehyde**	40	1.489	**0.684**	0.581	0.383	0.000
	23,599	1.500	0.580	**0.673**	0.273	0.000
**OSL7**	35	1.007	**0.648**	0.295	0.128	0.000
	1171	1.285	**0.702**	0.445	0.191	0.000
**C34**	187	1.142	**0.506**	0.205	0.137	0.512
	10,959	1.428	0.560	0.216	0.102	**0.903**
**JTT705**	439	1.033	**0.539**	0.391	0.188	0.010
	14,650	1.127	**0.592**	0.347	0.188	0.000

**Table 3:** Non LPS-like compounds identified with the TLR4/MD-2 antagonist activity. The calculated ADME parameters show the compounds have promising drug-like properties. The calculated logP is shown to illustrate the importance of a relatively high lipophilicity for the activity. ^a^ ChemScketch and ^b^ MolInspiration (www.molinspiration.com, accessed on 8 September 2022). Additional ADME parameters calculated with Swiss-ADME (http://www.swissadme.ch/, accessed on 8 September 2022) are ^c^ drug-likeness according to Lipinski’s rule and water solubility as ^d^ logS.

Compound	Structure	LogP ^a^	LogP ^b^	Drug-Likeness ^c^	LogS ^d^
ID-5382 (B)	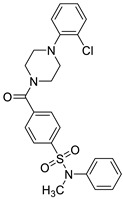	2.77 ± 0.49	4.13	Yes; 0 violations	−5.49
MS21 (F)	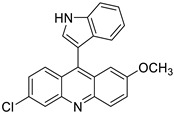	5.89 ± 0.40	6.331	Yes; 0 violations	−6.29
MS32 (H)	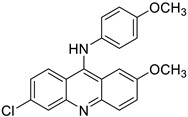	4.20 ± 0.83	6.434	Yes; 0 violations	−5.96
MS35 (I)	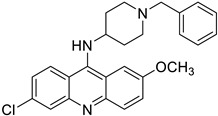	4.68 ± 0.84	6.728	Yes; 1 violation: MLOGP > 4.15	−6.37
MS45 (J)	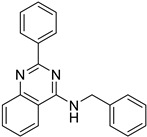	5.10 ± 0.45	5.712	Yes; 1 violation: MLOGP > 4.15	−5.54
PM1090 (X)	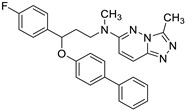	5.70 ± 0.89	6.063	Yes; 1 violation: MLOGP > 4.15	−6.48
PM1200 (Z)	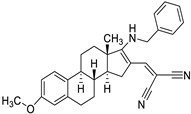	6.29 ± 0.45	6.116	Yes; 0 violations	−6.35
Sorafenib (M4)	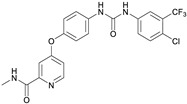	6.13 ± 0.36	5.89	Yes; 0 violations	−5.11

## Data Availability

Not applicable.

## Supplementary Materials

The following supporting information can be downloaded at: https://www.mdpi.com/article/10.3390/biomedicines10092326/s1, Figure S1: Top scoring compounds obtained by SBVS and LBVS on the Log P 1000 database. Figure S2: Top scoring compounds obtained by SBVS and LBVS on the SPECS database. Figure S3: Known antagonists of the MD-2 reported in the literature. Figure S4: Cell-based colorimetric assay for the detection of biological active endotoxin. Figure S5: Colchicine (left), pironetin (center) and euodenine A (right) chemical structures. Table S1: 2D description and the respective scores from ID-5382, AG-690/11203225 and AF-399/15128553.Table S2: 2D Chemical structure of predicted TLR4 modulators identified by computational drug repurposing, and kept for future structure similarity search.Table S3: 2D Chemical structure from PM databases obtained from SBVS. Table S4: 2D Chemical structure from JCM databases obtained from SBVS. Table S5: 2D Chemical structure from JRP and AM databases obtained from SBVS.
