# Supplementary material for: Small Molecules as Toll-like Receptor 4 Modulators Drug and In-House Computational Repurposing
Source: Biomedicines. 2022 Sep 19;10(9):2326. doi: 10.3390/biomedicines10092326 (PMC9496124; doi:10.3390/biomedicines10092326)
Supplement: Supplementary file 1 [file biomedicines-10-02326-s001.zip › biomedicines-1853568-supplementary materials.pdf]

Supplementary **Materials**

Small Molecules as Toll-like Receptor 4 Modulators. Drug and *in-house* Computational Repurposing

Lucía Pérez-Regidor [1,†], Joan Guzmán-Caldentey [1,†], Nils Oberhauser [1,2], Carmen Punzón [3], Balázs Balogh [4], José R. Pedro [5], Eva Falomir [6], Alessandra Nurisso [2], Péter Mátyus [4,‡], J. Carlos Menéndez [7], Belén de Andrés [8], Manuel Fresno [3] and Sonsoles Martín-Santamaría [1,*]

1 Department of Structural and Chemical Biology, Centro de Investigaciones Biológicas "Margarita Salas", CSIC, C/Ramiro de Maeztu, 9, 28040 Madrid, Spain

2 School of Pharmaceutical Sciences, University of Geneva, University of Lausanne, Rue Michel Servet 1, CH-1211 Geneva, Switzerland

3 Centro de Biología Molecular "Severo Ochoa", CSIC-Universidad Autónoma de Madrid, 28049 Madrid, Spain

4 Department of Organic Chemistry, Semmelweis University, H ̋o gyes E. u. 7, H-1092 Budapest, Hungary

5 Department of Organic Chemistry, Universidad de Valencia, 46100 Valencia, Spain

6 Department of Inorganic and Organic Chemistry, Escuela Superior de Tecnología y Ciencias Experimentales, University Jaume I, Av. Sos Baynat, s/n, 12006 Castellón, Spain

7 Unidad de Química Orgánica y Farmacéutica, Departamento de Química en Ciencias Farmacéuticas, Facultad de Farmacia, Universidad Complutense, 28040 Madrid, Spain

8 Immunobiology Department, Carlos III Health Institute, 28220 Madrid, Spain

* Correspondence: smsantamaria@cib.csic.es

† These authors contributed equally to this work.

‡ Current address: E-Group ICT Software Zrt., Kacsa utca 11, H-1027 Budapest, Hungary.

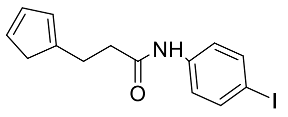

Compound 152

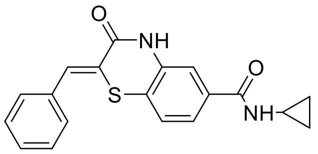

Compound 568

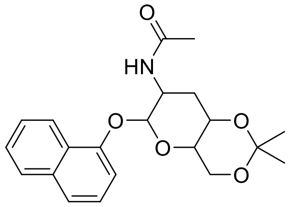

Compound 383

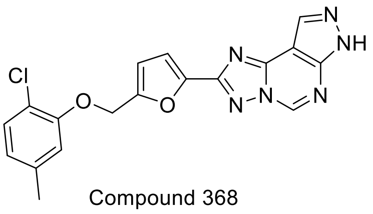

Compound 368

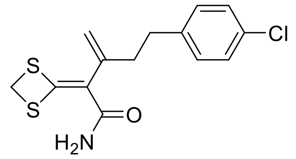

Compound 492

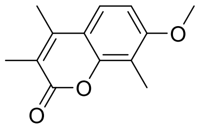

Compound 42

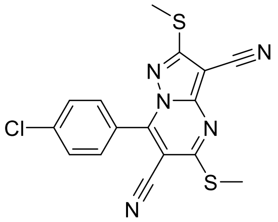

Compound 138

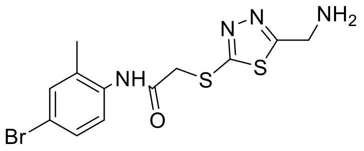

Compound 575

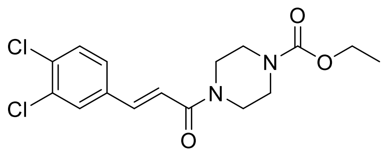

Compound 548

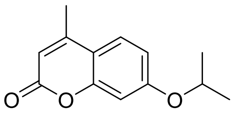

Compound 46

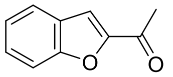

Compound 40

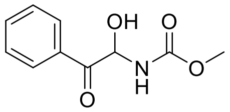

Compound 35

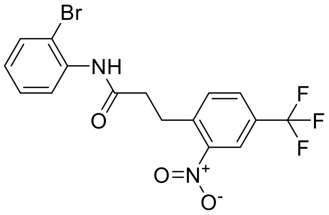

Compound 187

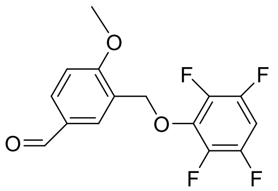

Compound 439

Figure S1. Top scoring compounds obtained by SBVS and LBVS on the Log P 1000 database.

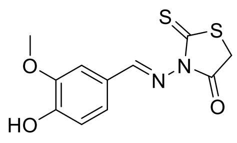

Compound 481

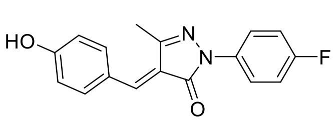

Compound 19907

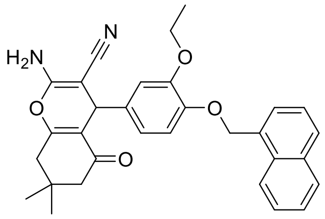

Compound 20513

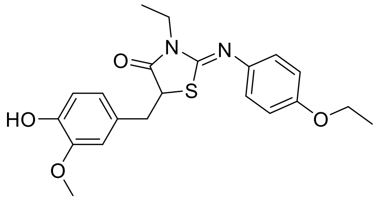

Compound 20700

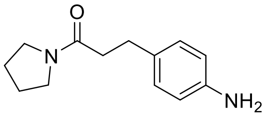

Compound 21315

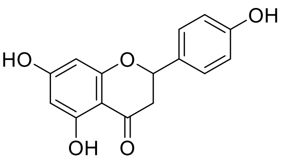

Compound 120

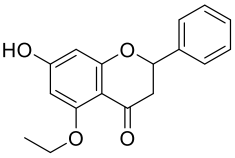

Compound 28

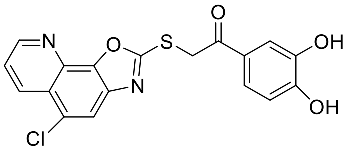

Compound 22298

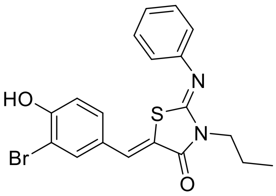

Compound 23010

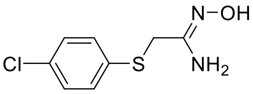

Compound 3203

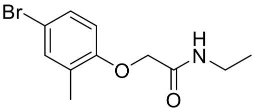

Compound 23599

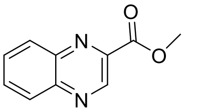

Compound 1171

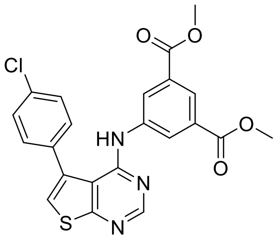

Compound 10959

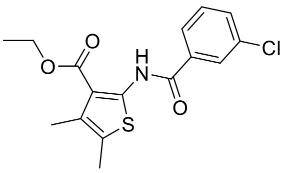

Compound 14650

Figure S2. Top scoring compounds obtained by SBVS and LBVS on the SPECS database.

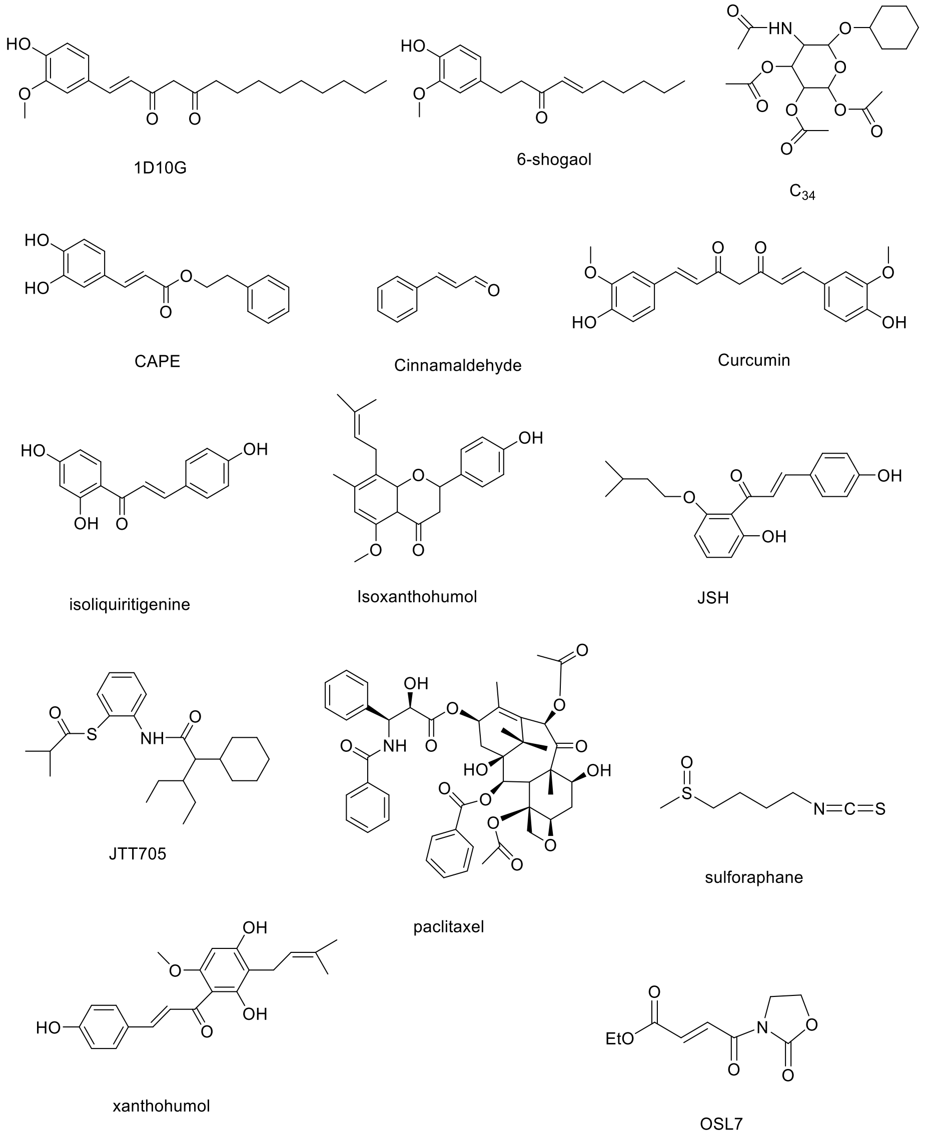

Figure S3. Known antagonists of the MD-2 reported in the literature.

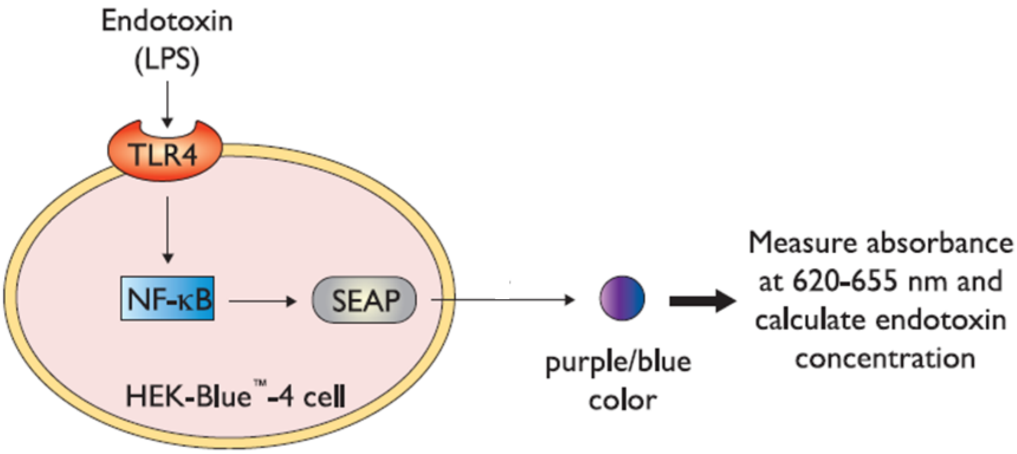

Figure S4. Cell-based colorimetric assay for the detection of biological active endotoxin (figure extracted from https://www.invivogen.com/hek-blue-lps-detection-kit).

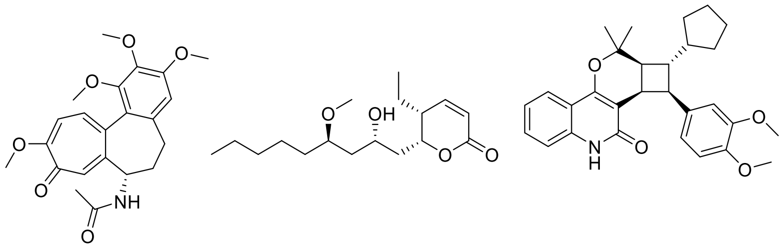

Figure S5. Colchicine (left), pironetin (center) and euodenine A (right) chemical structures.

Compound	Database	S-score	Structure
ID-5382	Log P 1000	1.231	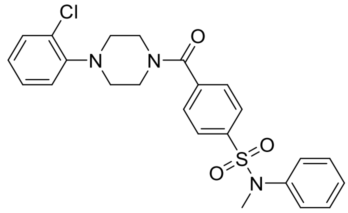
AG-690/11203225	SPECS	1.114	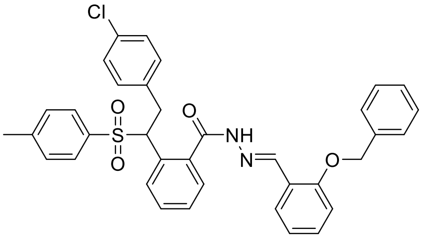
AF-399/15128553	SPECS	1.074	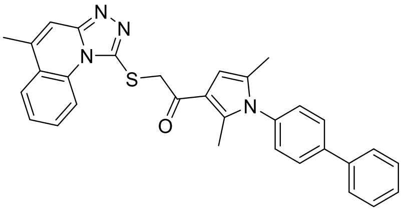

Table S1. 2D description and the respective scores from **ID-5382**, **AG-690/11203225** and **AF-399/15128553**.

Compound number	ZINC ID	Usual/Commercial name	2D structure
**146**	3830716	Diphenoxylate	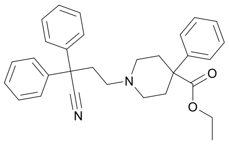
**157**	1493878	Sorafenib	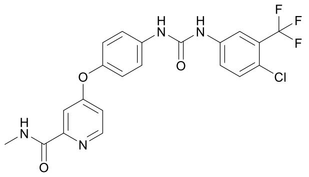
**177**	15919406	Ono-Rs 411	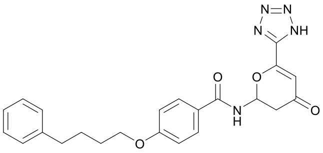
**179**	52509366	Zelboraf	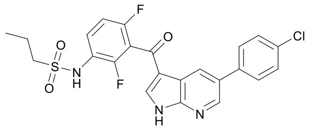
**208**	53073961	Antrafenine	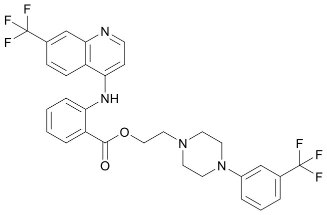
**212**	19685790	Lercanidipine	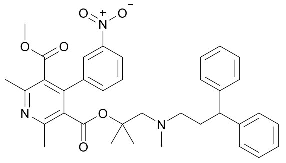

Table S2. 2D Chemical structure of predicted TLR4 modulators identified by computational drug repurposing, and kept for future structure similarity search.

Number	Compound	Structure
**1**	PM1097_p_R/1097	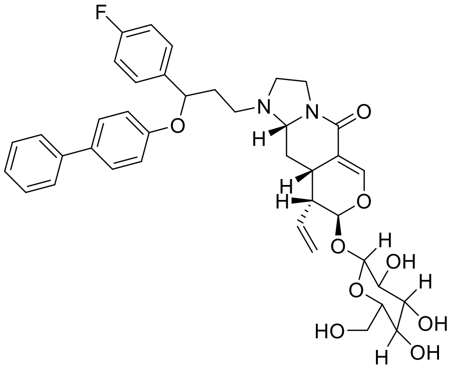
**2**	PM1811	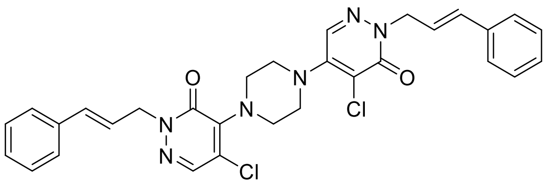
**3**	PM1779	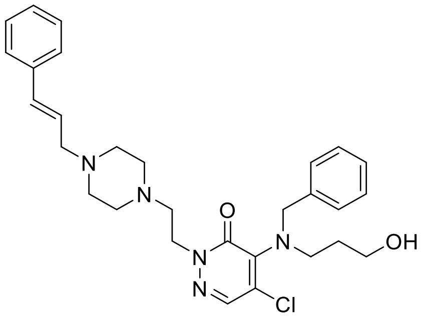
**4**	PM567S ó R	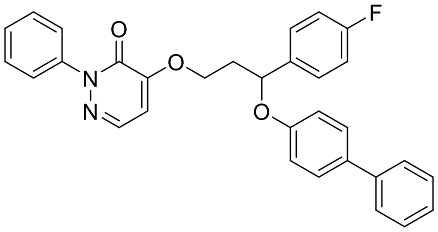
**5**	PM1090	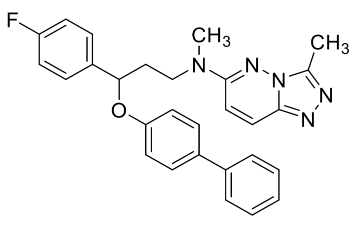
**6**	PM810	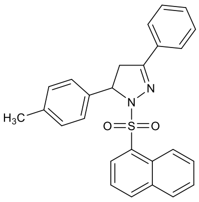

**7**	PM1758	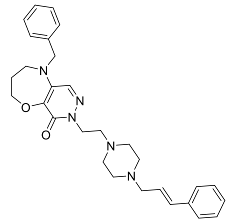
**8**	PM1200	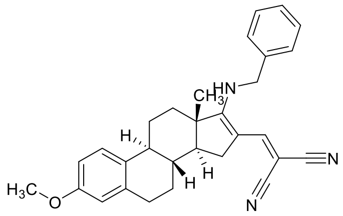

Table S3. 2D Chemical structure from PM databases obtained from SBVS.

Number	Compound	Structure
**1**	MS_35/35p	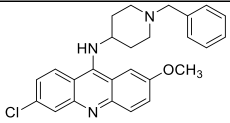
**2**	MS_29	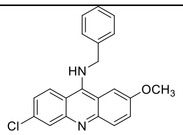
**3**	MS_40	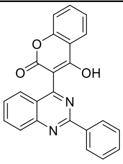
**4**	MS_34	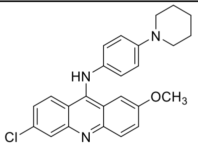
**5**	MS_31	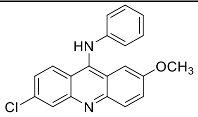
**6**	MS_22	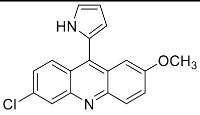
**7**	MS_45	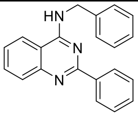
**8**	MS_21	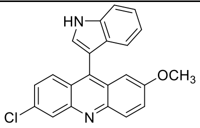
**9**	MS_32	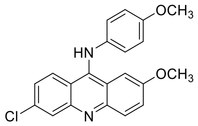
**10**	MS_26	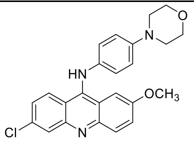
**11**	MS_14	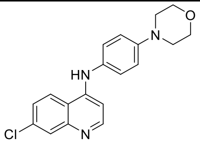

**12**	MS_49	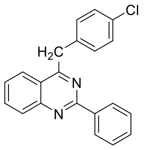
**13**	MS_37	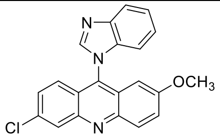
**14**	MS_46	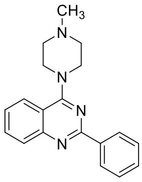
**15**	MS_20	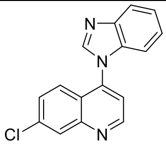

Table S4. 2D Chemical structure from JCM databases obtained from SBVS. Quinoline family: MS-**14**, MS-**20.** Quinazoline family: MS-**40**, MS-**45**, MS-**49.** Acridine family: MS-**32,** MS-**21,** MS-**35**, MS-**29,** MS-**22**, MS-**26**, MS-**31**, MS-**34**, MS-**37**

Name	Structure	Name	Structure
**JRP07**	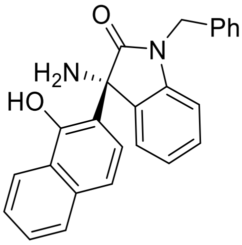	**AM15**	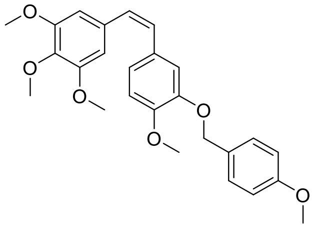
**JRP10**	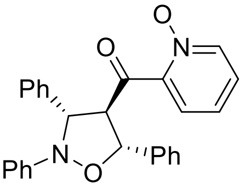	**AM18**	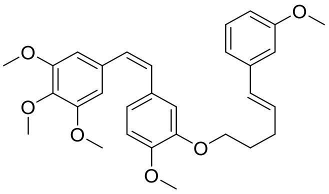
**JRP18**	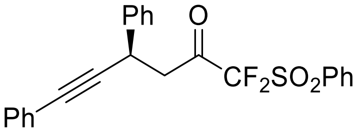	**AM19**	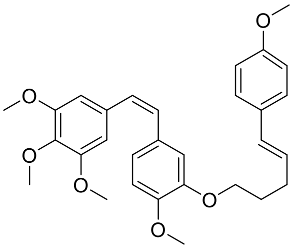
**AM20** **momo**	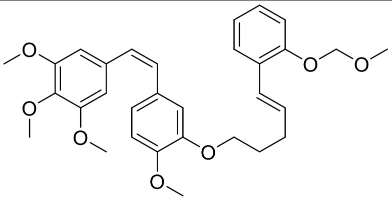	**AM54**	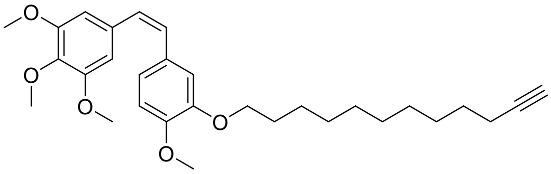
**AM21**	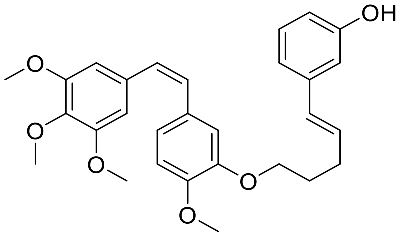	**AM57**	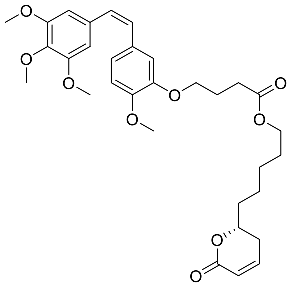
**AM20**	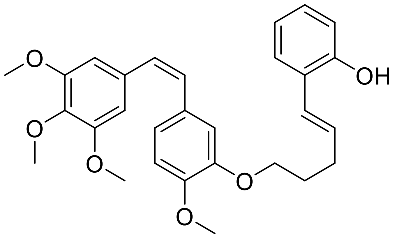	**AM58**	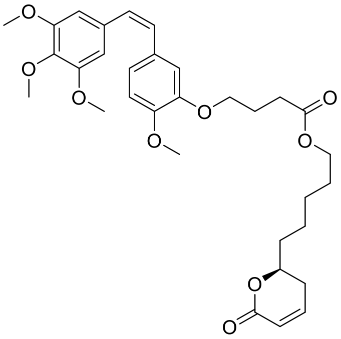
		**AM59**	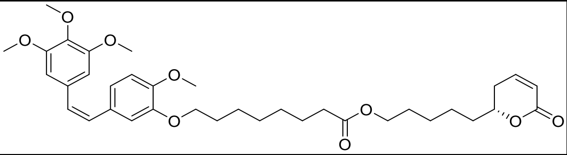

**AM22**	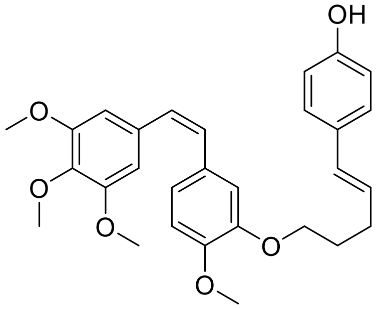	**AM62**	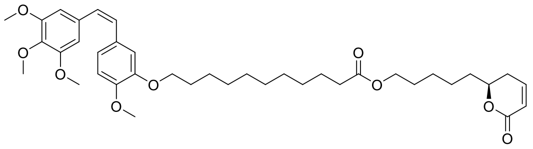
**AM23**	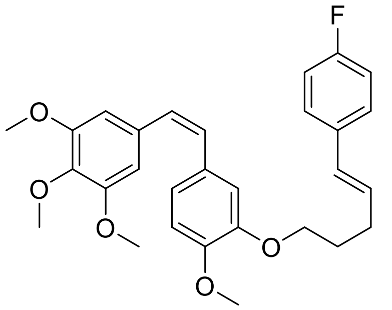	**AM65**	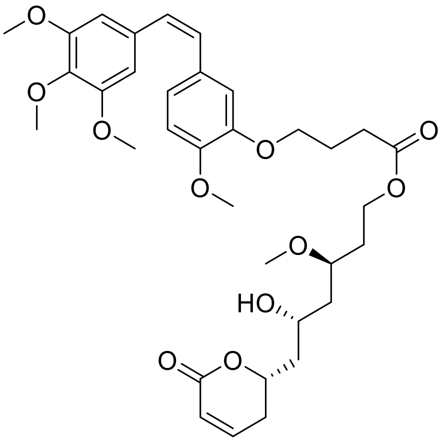
**AM24**	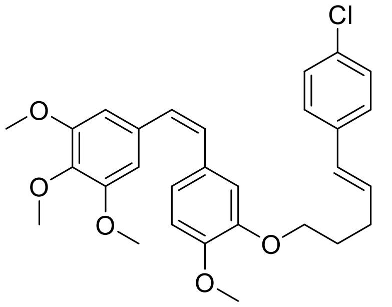	**AM66**	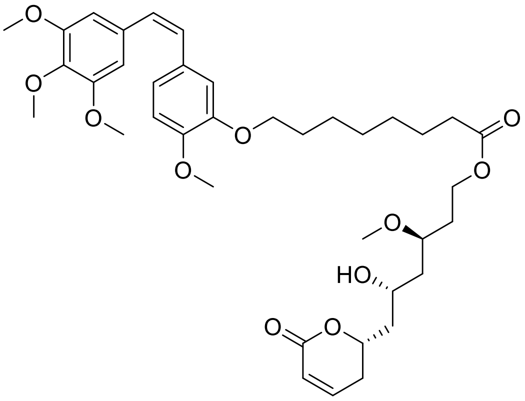
**AM25**	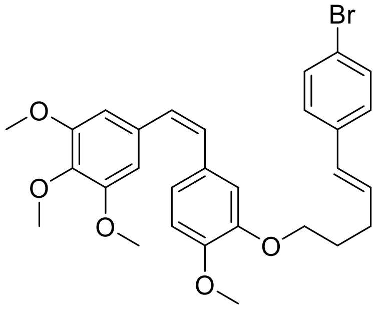	**AM72**	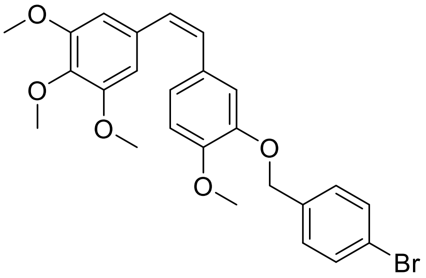

**AM40**	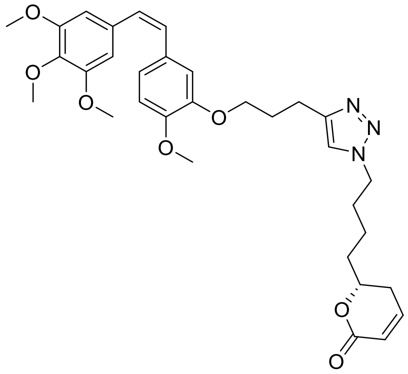	**AM16**	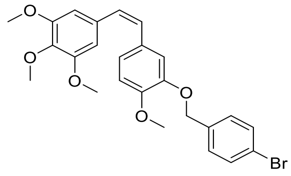
**AM41**	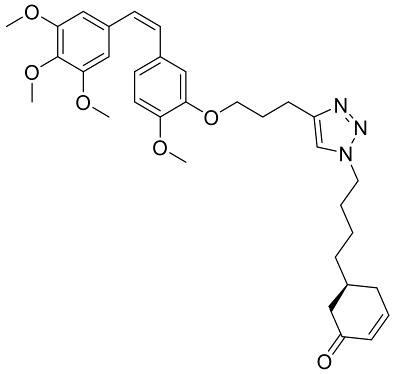	**JRP01**	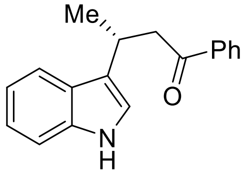
**AM42**	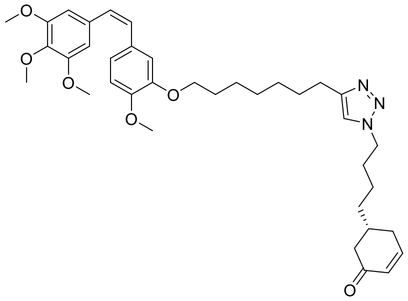	**AM08**	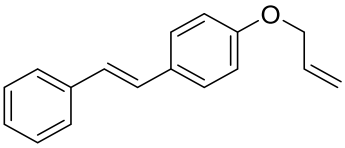
**AM48**	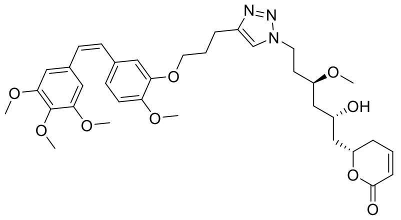	**AM14**	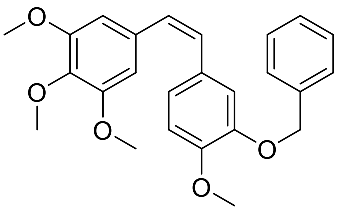
**AM53**	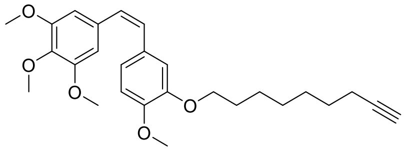	**AM71**	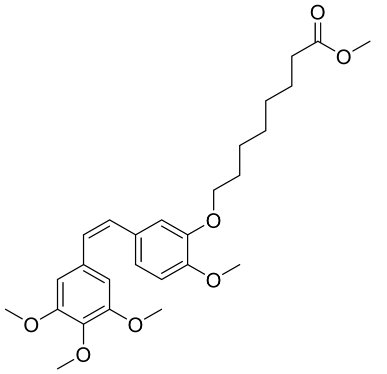

Table S5. 2D Chemical structure from JRP and AM databases obtained from SBVS.
